# Autosomal dominant Riggs-type congenital stationary night blindness with fundus sheen and retinal atrophy due to a novel *GNAT1* p.Gln200Arg variant

**DOI:** 10.1007/s10633-026-10100-2

**Published:** 2026-04-09

**Authors:** Jeremy J. Chou, Rachael C. Heath Jeffery, Jennifer A. Thompson, Samuel McLenachan, Enid S. Chelva, Tina M. Lamey, Terri L. McLaren, Fred K. Chen

**Affiliations:** 1 Ophthalmology, Department of Surgery, University of Melbourne, East Melbourne, Victoria Australia; 2 Centre for Ophthalmology and Visual Sciences (Lions Eye Institute), University of Western Australia, Nedlands, WA Australia; 3 Royal Victorian Eye and Ear Hospital, East Melbourne, Victoria Australia; 4 Department of Technology and Medical Physics, Sir Charles Gairdner Hospital, 2 Verdun Street, Nedlands, WA Australia; 5 Department of Ophthalmology, Royal Perth Hospital, Perth, WA Australia; 6 Department of Ophthalmology, Perth Children’s Hospital, Perth, WA Australia

**Keywords:** Inherited retinal disease, Electroretinography, Retinitis pigmentosa, CSNB, Nyctalopia, Transducin alpha-subunit

## Abstract

**Purpose:**

To report an Australian family with congenital stationary night blindness (CSNB, OMIM#139,330) harbouring a novel *GNAT1* c.599A > G (p.Gln200Arg) variant. In contrast to previous case reports we observed a fundus sheen, outer retinal changes and electrophysiological features of cone dysfunction in addition to a Riggs-type CSNB.

**Methods:**

Ophthalmic history, clinical examination and multimodal imaging including fundus autofluorescence (FAF) and optical coherence tomography (OCT) were obtained. Full-field electroretinography (ERG) was conducted according to the International Society for Clinical Electrophysiology of Vision (ISCEV) standards. Genetic testing was performed using a targeted next-generation sequencing panel with familial segregation confirmed by Sanger sequencing. In silico prediction tools and protein structural modelling were used to evaluate the pathogenicity and functional impact of the *GNAT1* p.Gln200Arg variant respectively.

**Results:**

Our proband, a 21-year-old transgender male, and his 62-year-old mother had a lifelong history of nyctalopia and visual acuity of 6/6 OU. The mother exhibited a golden sheen, sectoral chorioretinal atrophy and bone spicules. In the proband FAF imaging revealed hypoAF inferiorly with an arc of hyperAF whilst his mother had regions of hypoAF associated with outer retinal atrophy. Full-field electroretinography in the proband showed a cone-isolated retina with normal light-adapted responses. The mother had reduced and delayed light-adapted 30 Hz flicker. Both carried the *GNAT1* variant NM_000172.4:c.599A > G (p.Gln200Arg). In silico analysis predicted impaired GTPase activity in Arg200 and constitutively active signally after photoactivation. The c.599A > G variant was classified as likely pathogenic.

**Conclusions:**

Our study suggests the *GNAT1* p.Gln200Arg variant can manifest as both a Riggs-type CSNB and a rod-cone dystrophy within the same pedigree. This work expands the phenotypic spectrum of *GNAT1*-associated retinopathy and identifies *GNAT1* as another potential cause of a fundus sheen.

**Supplementary Information:**

The online version contains supplementary material available at 10.1007/s10633-026-10100-2.

## Introduction

Congenital Stationary Night Blindness (CSNB) refers to a group of inherited retinal diseases (IRDs) characterised by nyctalopia from birth [1]. Among the various subtypes, Riggs-type CSNB is defined by a normal fundus appearance with abnormal rod phototransduction resulting in a profoundly reduced dark-adapted (DA) 0.01 cd/m^2^ full-field electroretinography (ERG) response. The remaining DA3 and DA10 signals are derived from the dark-adapted cones [2, 3]. Pathogenic variants in *GNAT1*, which encodes the rod-specific transducin α-subunit, are one cause of autosomal dominant Riggs-type CSNB [2–4]. Until now, only three dominant pathogenic variants in *GNAT1* (p.Gly38Asp [French, Japanese] [5, 6], p.Ile52Asn [Chinese] [4] and p.Gln200Glu [Danish] [7]) have been reported in the literature. A fourth variant p.Asn251Lys, reported in two unrelated Korean families, remains a variant of uncertain significance [8, 9]. To date, all the dominant cases reported non-progressive features and a normal fundus appearance with no structural retinal abnormalities on multimodal imaging [4–8]. In this report, we describe a novel *GNAT1* variant NM_000172.4:c.599A > G (p.Gln200Arg) that manifested a fundus sheen and sectoral chorioretinal atrophy in addition to the electrophysiological features of a Riggs-type CSNB and a rod-cone dystrophy within the same pedigree.

## Methods

The study protocol adhered to the tenets of the Declaration of Helsinki and ethics approval was obtained from the Human Ethics Office of Research Enterprise, The University of Western Australia (2021/ET000151) and Sir Charles Gairdner Osborne Park Health Care Group Human Research Ethics Committee (RGS04985). Informed consent was obtained from both patients.

A 21-year-old transgender proband and his 62-year-old mother from an Australian-British family were evaluated for lifelong nyctalopia. Both received an ophthalmic assessment including best corrected visual acuity (BVCA) using the ETDRS chart, slit-lamp biomicroscopy and fundus examination. Multimodal retinal imaging included ultra-wide field pseudocolour and fundus autofluorescence (FAF) using the California (Optos PLC, Dunfermline, United Kingdom) and Heidelberg Spectralis (Heidelberg Engineering, Heidelberg, Germany) 55° or 30° blue wavelength FAF and spectral-domain optical coherence tomography (OCT).

Electrophysiological assessment, included electrooculography (EOG) and electroretinography (ERG), performed in accordance to the International Society for Clinical Electrophysiology of Vision (ISCEV) standards [10]. Full-field electroretinography (ffERG) included dark-adapted (DA0.01, DA0.3 Red, DA3, DA10 and DA oscillatory potentials) and light-adapted (LA3, LA30Hz) recordings. Additional ERG protocols such as On/Off bipolar ERG, photopic negative response (PhNR), multifocal ERG (mfERG) and pattern ERG were also performed.

Patient and familial DNA were collected through the Australian Inherited Retinal Disease Registry and DNA Bank. Genetic testing on the proband was performed by target enrichment (capture) and Next Generation Sequencing (NGS) using the MVL Vision Panel v19 (Molecular Vision Laboratory, Oregon, United States of America) consisting of 1,098 nuclear genes, the mitochondrial genome and copy number variation analysis. NGS achieved an average coverage of 500 reads and at least 30X coverage in > 96% of the panel (Supplementary Material). Whole exome sequencing was performed independently on the proband’s affected mother using the 3B-EXOME (3billion, Inc, Seoul, South Korea) which also detects small variants and copy number variations (Supplementary Material). Both MVL and 3 billion have College of American Pathologists (CAP) accreditation and meet the Clinical Laboratory Improvement Amendments (CLIA) requirements. Sanger sequencing was performed on the proband’s unaffected father (through MVL), affected mother’s sister and her two unaffected daughters (proband’s maternal cousins, in-house). Alignment and variant calling were performed using GRCh37 and adhered to ACMG guidelines [11]. Potential pathogenicity of variants was assessed in silico with different tools, including REVEL, CADD, Alpha Missense, PolyPhen2, SIFT and Mutation Taster.

The effect of the GNAT1 Gln200Arg substitution was modelled in ChimeraX (v.1.10) using the bovine GNAT1-GDP-AlF_4_ x-ray crystallographic model (template file: 1TAD.cif [12]). The bovine GNAT1 amino acid sequence was highly conserved with identical residue numbering to the human protein. The cif file was first converted to pdb format and additional copies of the complex were removed from the model to leave a single GNAT1-GDP-AlF_4_ monomer. Replacement of the Gln200 residue with Arg was performed using the Rotamers dialogue, with and without consideration of solvent molecules. 75 Gln200Arg rotamer substitutions (Dunbrack library) were evaluated for clashes. A single rotamer (Chi1 175.6; Chi2 176.5; Chi3 179.2; Chi4 179.1) was identified with no clashes with other protein residues and was used to generate the mutant protein. Seven additional rotamers were identified that showed proximity of the Arg sidechain with the catalytic water molecule (HOH 400), however, each of these showed multiple clashes with nearby residues and were rejected.

## Results

### The Proband (III:1)

**Fig. 1:**
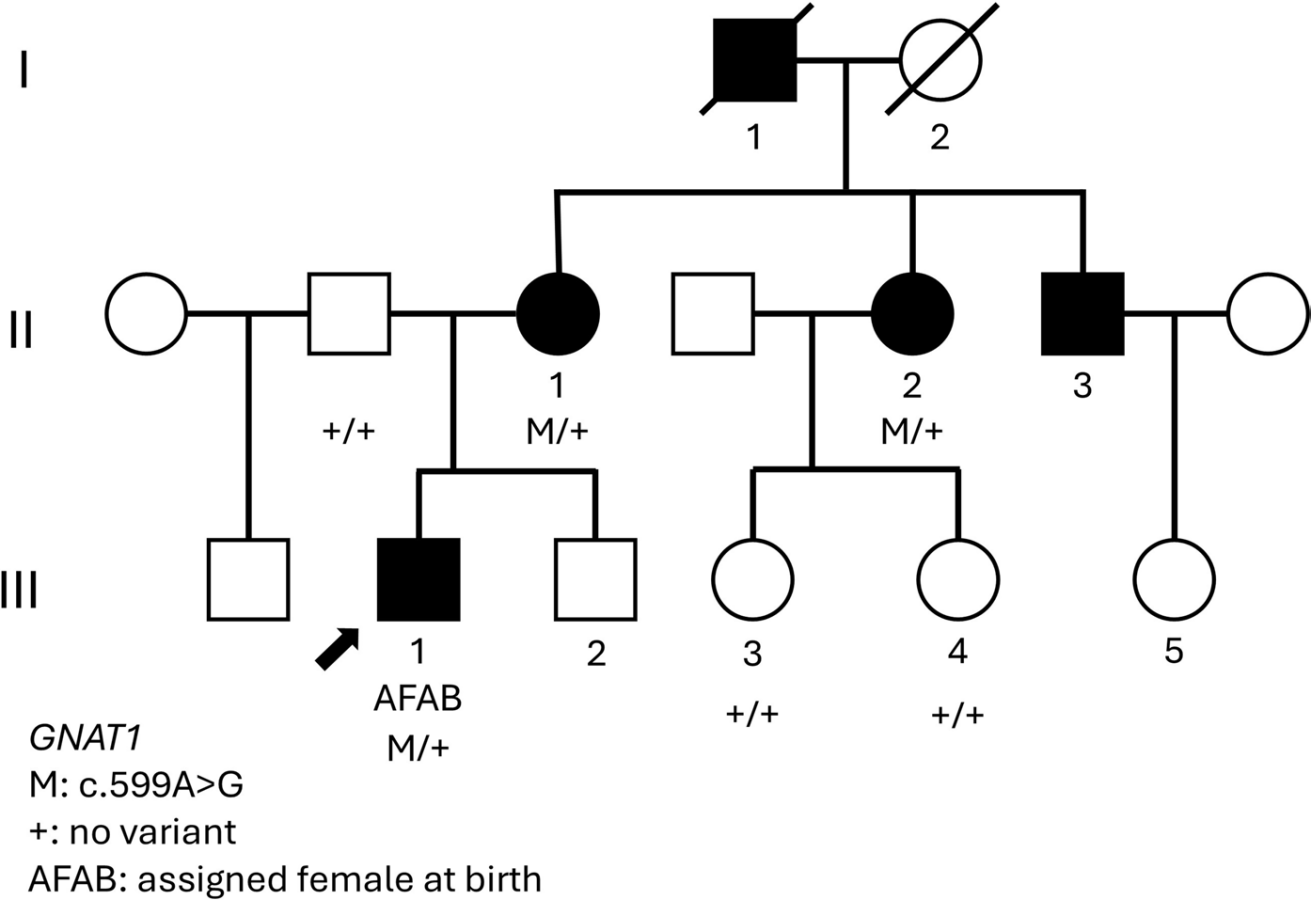
Pedigree chart showing the male proband (III:1) who was assigned female at birth and his mother (II:1) and aunty (II:2) were carriers of the *GNAT1* c.599A > G variant. Proband’s father and cousins (III:3 and III:4) do not carry this variant

**Fig. 2:**
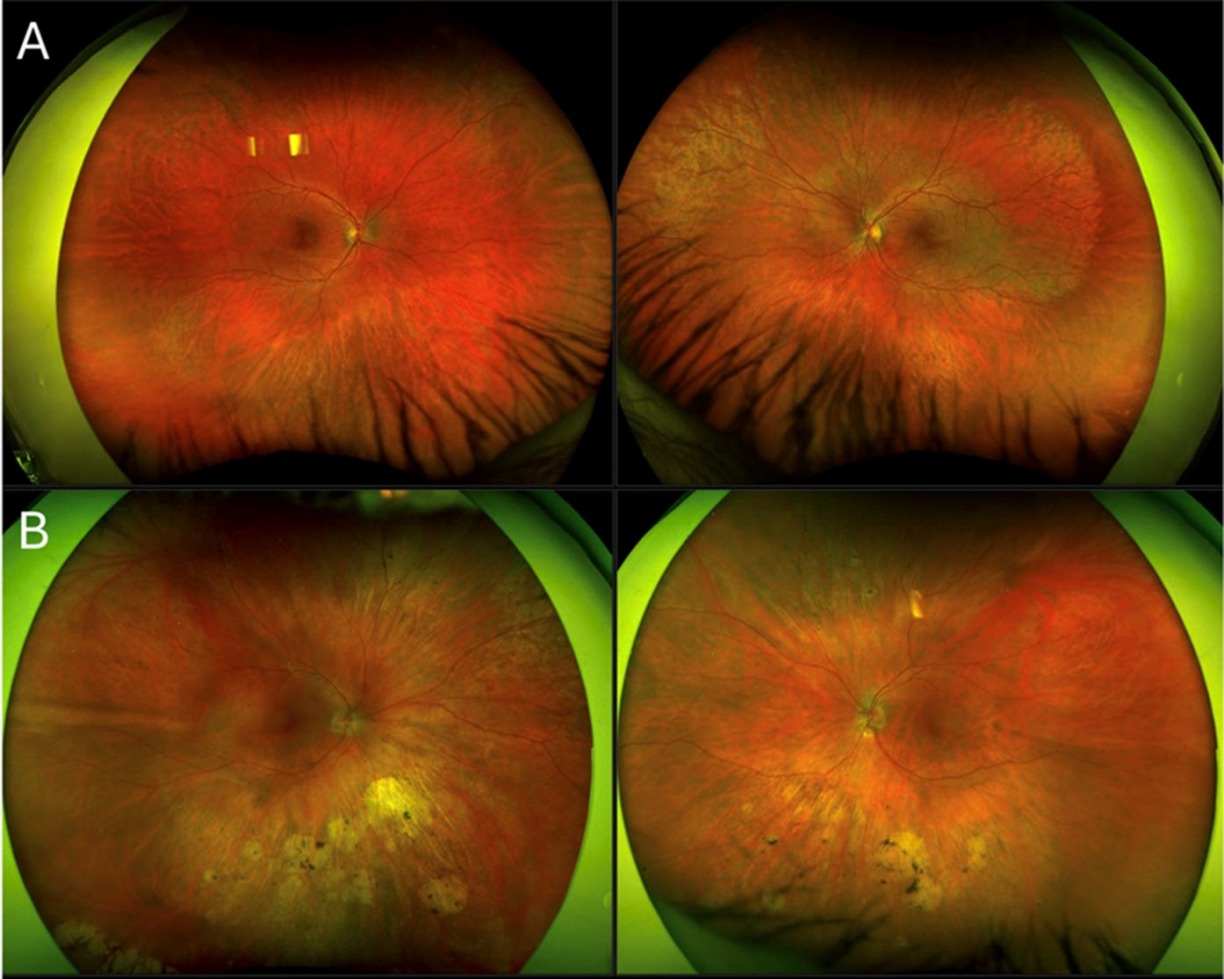
Optos pseudo-colour imaging of the proband (A) and his mother (B) showed a fundus sheen and retinal atrophy predominantly in the inferior perimacular region

**Fig. 3:**
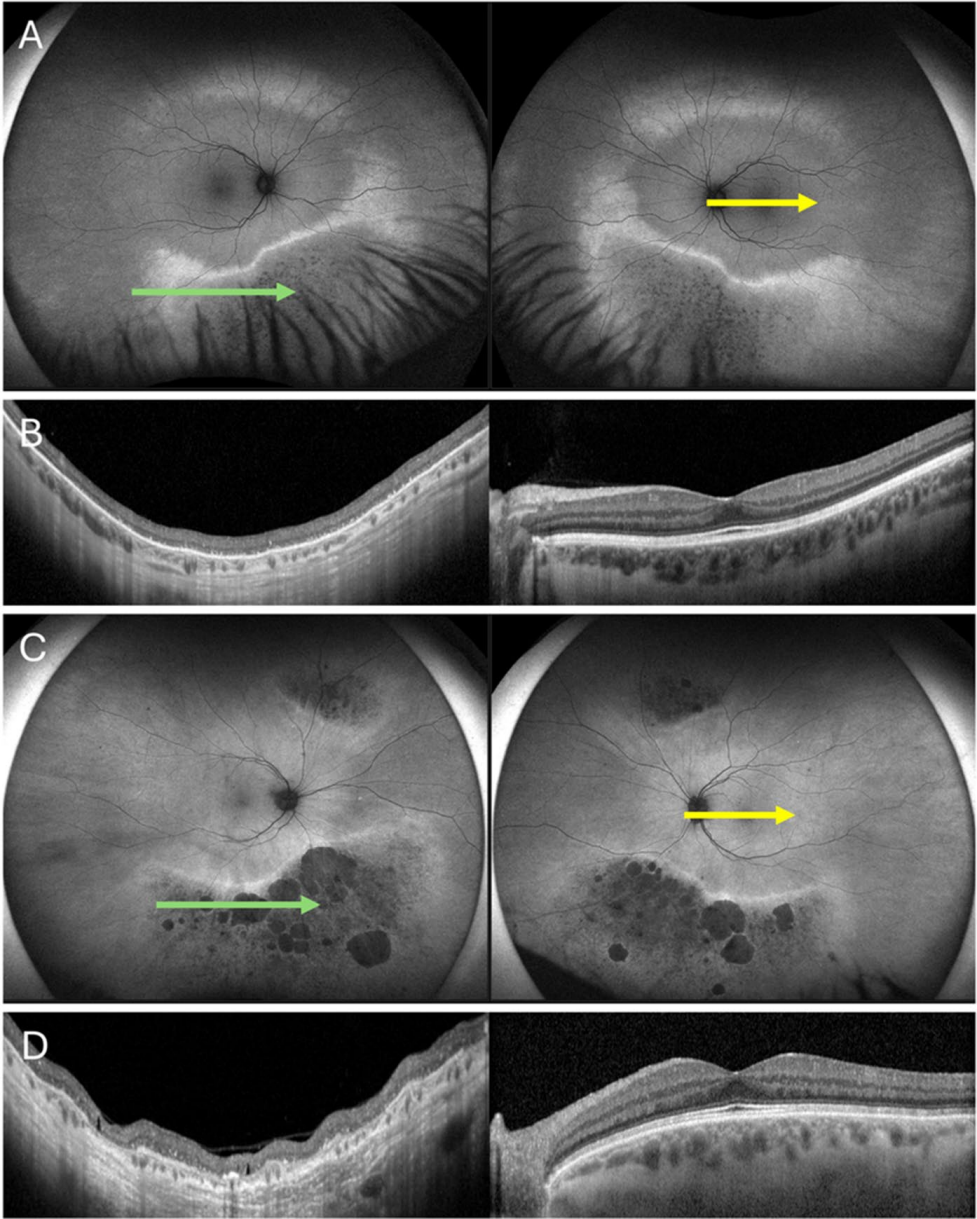
Optos fundus autofluorescence (FAF) and optical coherence tomography (OCT) of the proband (A, B) and his mother (C, D). FAF shows an arc of hyperAF around the macula from inferonasal to superotemporal retina (A). OCT shows loss of outer nuclear layer in the inferior retina (green arrow) and normal foveal architecture (yellow arrow) in the proband (B). The proband’s mother had more severe loss of FAF signal inferiorly and superonasally (C). There was corresponding outer retinal atrophy in regions of hypoAF despite preservation of foveal architecture on OCT (D)

Our 21-year-old transgender male (Fig. 1) reported lifelong nyctalopia with BCVA of 6/7.5 OD and 6/6 OS. Subjective refraction was -4.75/-1.25 × 5 OD and -4.50/-1.50 × 3 OS. Fundus examination showed a golden reflex in the nasal and temporal macular regions with inferior perimacular atrophy (Fig. 2A). FAF revealed bilateral inferior hypoAF regions with an arc of hyperAF nasally and superiorly extending to the equator (Fig. 3A). OCT revealed disruption of the outer retinal bands in areas of inferior hypoAF (Fig. 3B). Visual fields showed bilateral superior arcuate defects separate from the physiological blind spot.

**Fig. 4:**
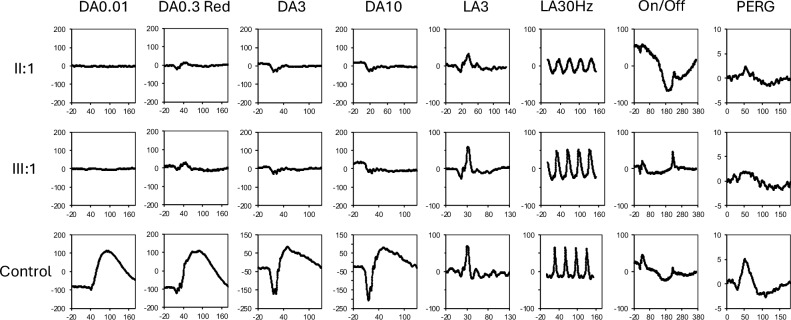
Full-field electroretinography (ERG) showing undetectable dark-adapted (DA) 0.01 response in both the proband (III:1) and his mother (II:1). The DA0.3 Red response showed preservation of the x-wave arising from dark-adapted cones and absence of the b-wave which indicates absence of rod function. The DA3 and DA10 both showed reduced a-wave with pseudonegative morphology due to dark-adapted cone contribution to the response. Light-adapted (LA) 3 response is normal in the proband but reduced in b-wave resulting in a b:a ratio of < 3.0 in the mother. The LA30Hz was normal in the proband but subnormal and delayed in the proband’s mother indicating secondary cone degeneration consistent with a mild rod-cone dystrophy. On/Off response demonstrated additional on-bipolar cell dysfunction in the proband’s mother. Pattern ERG was within normal range although the P50 component amplitude was low

Full-field ERG showed an undetectable DA0.01 response in both eyes. The DA3 responses showed reduced a- and b-waves with a reduced b:a ratio of 0.6(normal > 1.2). The DA10 showed an even lower b:a ratio of 0.2. The DA3 and DA10 signals arise from the dark-adapted cones as demonstrated by the preservation of DA0.3 Red x-wave. The LA3 and LA30Hz were within normal limits (Fig. 4). On/Off ERG and PhNR responses were bilaterally normal. The multifocal ERG showed a marginal reduction in the response densities within the central 10 degrees. Pattern ERG P50 amplitude was 2.2–2.8 µV, within the normal range (2.0–9.0 µV).

### Proband’s mother (II:1)

The proband’s 62-year-old mother (Fig. 1) also had as lifelong history nyctalopia with BCVA of 6/6 in both eyes. Family history revealed that her sister, brother and father were also night blind. Her refraction was + 0.75 DS (post-corneal refractive surgery) OD and -1.25/-1.75 × 85 OS. Fundus examination showed a superonasal retinal sheen, with prominent chorioretinal atrophy and bone spicules in the inferio nasal and superonasal periphery (Fig. 2B). FAF revealed multifocal round hypoAF regions inferiorly bordered by a hyperAF margin (Fig. 3C). These regions corresponded to severe outer retinal layer and choroidal atrophy on OCT (Fig. 3D).

Full-field ERG showed an undetectable DA0.01 response in both eyes. The DA3 responses showed reduced a- and b-waves with a reduced b:a ratio of 0.7 (normal > 1.2). The DA10 showed an even lower b:a ratio of 0.5. Similarly to the proband these signals arise from the dark-adapted cones as demonstrated by the preservation of DA0.3 Red x-wave. The LA3 showed reduced and delayed b-waves with b:a ratio of 2.7 (normal > 3.0) and normal a-wave (amplitude of 18 [normal: 14–38] µV with peak time of 16 [normal: 13–17] ms). The LA30Hz showed reduced (50 [normal 52–116] µV) and delayed (34 (normal: 25.0–29.5) ms) peaks. The On/Off ERG showed a mild reduction in b-wave amplitude at 20 (normal: 28–63) µV (Fig. 4). The PhNR responses were normal. Multifocal ERG showed reduced response densities in the inferior paramacular region. Pattern ERG P50 amplitude was 2.5–3.7 µV, within the normal range (2.0–7.0 µV).

### The proband’s aunty (II:2)

There was a life-long history of nyctalopia with a normal fundal examination and multimodal imaging. However, she had not undergone electrophysiological testing. Her two daughters were asymptomatic with normal retinal findings.

#### *Molecular genetic analysis and *in silico* modelling*

A novel heterozygous missense variant in *GNAT1*, namely NM_000172.4 (GNAT1): c.599A > G p.(Gln200Arg) was identified in the proband (III-1) and subsequently confirmed by Sanger sequencing (MVL) and an independent whole exome sequencing (3 billion) in his mother (II-1). In-house Sanger sequencing confirmed his maternal aunty (II-2) also carried the same variant (Supplementary Material). This variant was absent from the proband’s father’s DNA (MVL specific mutation analysis) and the aunty’s two daughters by in-house Sanger sequencing (Supplementary Material). 

The *GNAT1* c.599A > G variant was not observed in the Genome Aggregation Database (gnomAD) v4.1.0. In silico prediction tools (REVEL, Alpha Missense, PolyPhen2, CADD, SIFT, Mutation Taster) predict this variant to be damaging. In addition to the *GNAT1* variant, three other variants of uncertain significance (VUS) were found in the MVL Vision panel: *BBS9* c.736C > T (heterozygous), *TYR* c.1205G > A (heterozygous) and *TYR* c.1509G > C (heterozygous, see Supplementary Material). Given the absence of Bardet-Biedl syndrome or oculocutaneous albinism, these variants were not considered further. 

**Fig. 5:**
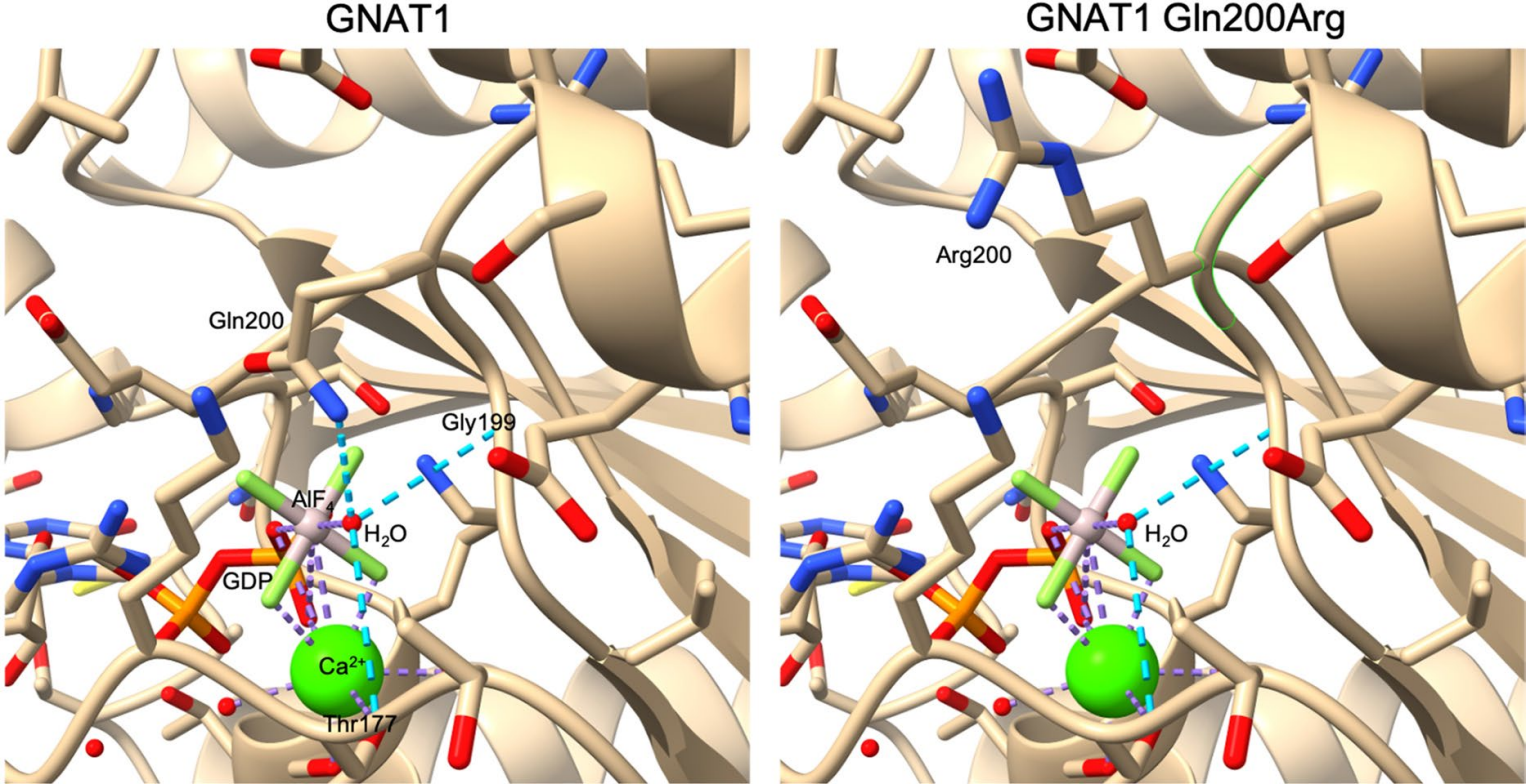
Protein modelling of GNAT1 Gln200Arg. The left panel shows the crystal structure of the GTPase catalytic site in the bovine GNAT1-GDP-AlF_4_ complex. The positions of residues forming hydrogen bonds (blue dashed lines) with the catalytic water molecule (H_2_O) are indicated. The AlF_4_ (AIF) and GDP molecules model the transition state of the GTP during hydrolysis while a calcium ion (Ca^2+^) models the Mg^2+^ ion that would be present in a physiological state. The right panel shows the structural model generated for the Gln200Arg substitution. The substitution results in movement of the Arg side chain away from the catalytic site, resulting in loss of hydrogen bonding with the catalytic water molecule

To investigate the effects of the GNAT1 Gln200Arg substitution, we utilized a bovine GNAT1-GDP-AlF_4_ crystal structure [12]. In this model, the Gln200 (homologous with human Gln200), Gly199 and Thr177 residues form hydrogen bonds with the catalytic H_2_O molecule (Fig. 5), stabilising its position to enable nucleophilic attack and cleavage of the Pγ–O bond of the bound GTP molecule (represented by the AlF_4_ molecule in the GNAT1-GDP-AlF4 model). This precise positioning of the catalytic H_2_O by Gln200 is critical for GTPase activity.

Evaluation of 75 Gln200Arg rotamers revealed a single rotamer that did not produce clashes with local residues, which was used to generate the GNAT1 Gln200Arg model shown in Fig. 5. This mutant GNAT1 model indicated that substitution of Gln200 with Arg results in the movement of the sidechain away from the catalytic site, disrupting hydrogen bonding with the catalytic H_2_0 (Fig. 5). The loss of interaction between residue 200 and the catalytic H_2_O would likely compromise the GTPase activity of GNAT1, creating a hyperactive mutant protein with reduced capacity to terminate signalling via GTP hydrolysis.

## Discussion

Our proband demonstrated a cone isolated retina whereby his rod function was absent and the DA3 and DA10 responses were generated from the dark-adapted cones giving rise to a pseudo-electronegative profile [13]. A true electronegative ERG, as seen in Schubert-Bornschein CSNB, exhibits a normal a-wave amplitude. In contrast, the smaller a-wave in the DA3 and DA10 responses originates from dark-adapted cones as confirmed by the preservation of an x-wave with the DA0.3 Red stimulation. The dark-adapted cones stimulate bipolar cells to generate a b-wave response. The decreasing b:a ratio (0.6 to 0.2 in proband and 0.7 to 0.5 in proband’s mother) with increasing flash luminance from 3 and 10 cd/m^2^ is consistent with the photopic hill phenomenon [14]. Together, these findings were consistent with Riggs-type CSNB and other causes of a cone isolated retina [13]. The proband’s mother, however, also exhibited impaired cone pathway responses in addition to absent rod-derived responses. A reduced b:a ratio in the LA3 and the reduced On-bipolar cell response indicated that she had a post-phototransduction cone pathway abnormality which may be due to transsynaptic bipolar cell degeneration secondary to cone dysfunction. However, the LA3 a-waves parameters were within normal limits. The presence of chorioretinal atrophy in this family suggest the Glu200Arg substitution may impact rod photoreceptor survival in addition to the desensitisation of the rhodopsin kinetics as seen in the stationary Riggs-type CSNB.

To date, five families carrying four variants in *GNAT1* have been described to exhibit an autosomal dominant-CSNB ERG phenotype (adCSNB, OMIM#139,330) (Table 1) [4–7, 9]. In contrast to our case, all reported cases, with one exception, demonstrated a normal fundus appearance; the exception being the Danish p.Gln200Glu pedigree. In this pedigree, one case presented with an RP phenotype (extinguished ERG) which the authors attributed to a coincidental, at the time undiscovered, RP gene [7]. However, they did not use ultrawide field FAF imaging to exclude subtle hypoAF changes in their younger affected family members to look for mild degeneration which may have been missed on clinical examination. Of note, ultrawide field imaging has not been used to examine the Nougaret or the Danish *GNAT1*-associated adCSNB pedigrees. In the original description of the Nougaret pedigree (c.113G > A), ERG showed absence of rod a-wave (dim blue flash) as opposed to Schubert-Bornschein type CSNB which has a normal rod a-wave [15]. They also demonstrated a biphasic a-wave and preserved b-wave with a dim white flash which they interpreted as evidence of residual rod function. Although the LA30Hz response was normal, the third cone-mediated oscillation of the 0.5 Hz red flash was lost and the dark-adaptation curve showed mild loss of sensitivity in the cone plateau indicating subtle cone dysfunction. In a subsequent Japanese study of the Nougaret-type adCSNB variant (c.113G > A), they demonstrated similar ERG features to our proband; undetectable rod response (DA0.01), uniphasic and broadened DA3 a-wave, b:a ratio of less than 1 in DA3 and DA10 with normal LA3 and LA30Hz cone responses [6]. The Chinese-type adCSNB variant (c.155 T > A) exhibited ERG features that were also similar except for the b:a ratio of > 1.0 in the DA3 response [2, 4]. As the authors did not report findings of the DA10, we were unable to assess whether a further reduction of the b:a ratio occurred with increasing flash luminance. The Danish-type adCSNB family reported by Szabo et al. described very similar features to our study with an absent rod b-wave in the scotopic ERG and a cone-like response to bright flashes. Interestingly, they reported normal-to-moderately decreased amplitudes in LA responses without specifying whether this was observed in LA3 or LA30Hz. One patient of this family (II-2) was reported to have an extinguished ERG without further clarification. The photopic ERG may not be sensitive enough to pick up secondary early-stage cone degeneration. Our findings of additional post-phototransduction cone system dysfunction in the mother of the proband suggests the possibility of a slowly progressive degeneration in dominant *GNAT1*-associated retinopathy as reported in the Danish pedigree.

**Table 1:** Clinical and genetic characteristics of patients with reported dominant pathogenic *GNAT1* variants

Nationality	Number of subjects: Affected; Examined; Generations	Sequence	Protein	Pathogenicity prediction*	Progressive	Refraction	Fundus appearance	ERG: rod function	ERG: cone function	References (year)
French	135 affected 29 examined 12 generations	c.113G > A	Gly38Asp	Likely pathogenic	No	Not measured	Normal	Residual rod function	Normal 30-Hz flicker‡	Dryja et al. [5] Sandberg et al. [15]
Japanese	8 affected 7 examined 3 generations	c.113G > A	Gly38Asp	Pathogenic	No	Mild myopia	Normal	Undetectable	Normal	Hayashi et al. [6]
Chinese	2 affected 2 examined 2 generations	c.155 T > A	Ile52Asn	Likely pathogenic	No	Mild myopia	Normal	Undetectable	Normal	Zeitz et al. [4] Marmor et al. [2]
Danish	9 affected 5 examined 4 generations	c.598C > G	Gln200Glu	Likely Pathogenic	No	Mild to severe myopia	Normal†	Undetectable	Normal to moderately reduced	Szabo et al. [7]
Australian	4 affected 2 examined 3 generations	c.599A > G	Gln200Arg	Likely pathogenic	Yes	Moderate myopia	Retinal sheen and atrophy	Undetectable	Normal to mildly reduced / delayed (proband’s mother)	This study

*ERG* electroretinography

^†^One case of retinitis pigmentosa (RP) carrying the same familial variant but the RP case was attributed to another gene

^‡^Impaired cone sensitivity during dark-adaptation

^*^Assigned by Leiden Open Source Variation Database (LOVD)

Clinical examination in the proband and his mother showed a peripheral fundus sheen which is a feature of Oguchi disease (CSNB1; MIM#258,100 & CSNB2; MIM#613,411), X-linked retinoschisis (XLRS1; MIM#312,700), *RPGR*-associated retinopathy (MIM#304,020, MIM#300,029) and *TTLL5*-associated retinopathy (MIM#615,860). More recently, a golden sheen was also observed in a Japanese family with a homozygous in-frame deletion causing recessive *GNAT1*-associated retinopathy [16]. In contrast, previous reports of recessive *GNAT1* disease by three other groups did not show a golden sheen in conjunction with either CSNB [17] (homozygous missense variant) or rod-cone dystrophy (homozygous truncating variant) phenotypes [18, 19]. The proposed mechanism of the golden sheen in Oguchi disease is a buildup of light-activated rhodopsin in the outer segments secondary to impaired inactivation by rhodopsin kinase (*GRK1*) or arrestin (*SAG*). Therefore, the mechanism of the golden sheen in Glu200Arg and Lys273del may be similar to Oguchi disease where activated rhodopsin accumulates due to the inability of alpha-transducin to hydrolyse and release the bound GTP molecule.

The similarities in phenotype between p.Gln200Glu and p.Gln200Arg may be explained by their common effects on the phototransduction pathway. Codon 200 lies within the switch II region of GNAT1 α-transducin known as the GTP-binding pocket and is essential for phototransduction termination as the alpha-subunit hydrolyses the GTP and converts it to GDP. Protein modelling of the Gln200Arg substitution in GNAT1 showed the Arg residue can be accommodated in the GTPase binding pocket without inducing steric clashes and that the mutant protein is likely to have compromised GTPase activity, which could lead to prolonged or constitutively active signalling after photoactivation. Previous in silico protein modelling of the GNAT1 Gln200Glu and Gln200Lys substitutions showed similar results, with movement of the sidechains away from the catalytic site [7]. Similarly, the Gln200Lys mutant protein has been shown to have reduced GTPase activity in biochemical assays, indicating repositioning of the residue 200 sidechain leads to loss of GTPase activity [20].

Limitations include our small sample size (n = 2) and lack of electrophysiology testing in the proband’s aunty who also carried the same familial variant. We have not performed functional validation of the *GNAT1* variant p.Gln200Arg identified in our two cases using a patient-derived cell model. While the golden sheen resembled Oguchi disease, we were unable to demonstrate reversibility of the fundus sheen on colour photography, OCT outer retinal band changes or rod dysfunction with overnight dark-adaptation. Examination of more individuals with the same *GNAT1* variant will allow for a more convincing genotype–phenotype correlation. Whilst an independent whole exome sequencing performed in the mother of the proband failed to show another genetic cause to explain her rod-cone dystrophy, we acknowledge a missed deep intronic or other non-coding variant(s) may account for the more severe phenotype. 

Our findings indicate that the novel Gln200Arg substitution is associated with an autosomal dominant pedigree presenting with a Riggs-type CSNB in our younger proband and a rod cone dystrophy in the proband’s mother. This report expands the phenotypic spectrum of dominant *GNAT1*-associated disease and highlights the importance of including dominant *GNAT1* disease in the differential diagnosis of a fundus sheen and late-onset rod-cone dystrophy.

## Supplementary Information

Below is the link to the electronic supplementary material.

Supplementary file1 (PDF 958 KB)

## Data Availability

All data supporting the findings of this study are available within the paper.
