# Supplementary material for: Autosomal dominant Riggs-type congenital stationary night blindness with fundus sheen and retinal atrophy due to a novel GNAT1 p.Gln200Arg variant
Source: Doc Ophthalmol. 2026 Apr 9;152(3):381–91. doi: 10.1007/s10633-026-10100-2 (PMC13194308; doi:10.1007/s10633-026-10100-2)

Supplementary Material

Autosomal dominant Riggs-type congenital stationary night blindness with fundus sheen and retinal atrophy due to a novel *GNAT1* p.Gln200Arg variant

1. MVL Vision Panel and variants

2. 3B-EXOME Information

3. Chromatogram from in-house Sanger

MVL Vision Panel

TECHNIQUES: Direct testing for pathogenic variants in the genes of the MVL Vision Panel v19 (https://www.molecularvisionlab.com/mvl-vision-panel/) was performed by target enrichment (capture) and Next Generation Sequencing. Our MVL Vision Panel (v19) consists of 1098 genes plus the mitochondria genome and mitochondria nuclear genes with an average coverage of ~500 reads and at least 30X coverage in >96% of the panel. Targeted regions cover all exons, exon-intron boundaries, and relevant, deep-intronic regions. Identified pathogenic variants related to patient conditions were confirmed by Sanger sequencing. All exons and exon/intron boundaries were sequenced. Exon 1 is defined as the exon having the start codon ATG. Codon 1 corresponds to the start ATG and nucleotide 1 to the A. Alignment and variant calling is done using GRCh37. Variants with dbSNP allele frequency >20% or previously identified as false positives are excluded. ACMG guideline was followed for our variant calling (Ref: Genet Med. 2015 May;17(5):405-24). The Sanger sequencing step was performed at NeoGenomics (Fort Myers, FL. CLIA # 45D1021782). Primer designs and PCR reactions were performed at MVL. The NGS sequencing

Table 1. Pathogenic variants and variants of uncertain significance (VUS) identified in the patient.

#Gene	HGVSCoding	HGVSProtein	Rs	ExACAF	Zygosity	Pathogenicity
BBS9	NM 198428.3:c.736C>T	NP 940820.1:p.Leu246Phe	rs140821420	1.24E-04	hetero	VUS
GNAT1	NM_000172.4:c.599A>G	NP_000163.2:p.Gln200Arg	N/A	N/A	hetero	Likely pathogenic
TYR	NM 000372.5:c.1205G>A	NP 000363.1:p.Arg402Gln	rs1126809	0.176	hetero	VUS
TYR	NM_000372.5:c.1509G>C	NP_000363.1:p.Lys503Asn	rs138750983	5.27E-04	hetero	VUS

https://www.molecularvisionlab.com/mvl-vision-panel/

List of genes

Gene list: AARS2, AASS, ABAT, ABCA1, ABCA4, ABCB6, ABCB7, ABCC6, ABCD1, ABHD12, ACACA, ACACB, ACAD9, ACADL, ACADM, ACADS, ACADVL, ACAT1, ACBD5, ACO2, ACVR2B, ADAM9, ADAMTS10, ADAMTS17, ADAMTS18, ADAMTS2, ADAMTSL4, ADGRA3, ADGRV1, ADIPOR1, AFG3L2, AGBL5, AGK, AGPS, AGRN, AHI1, AHR, AIFM1, AIPL1, AKR1C1, ALAS2, ALDH18A1, ALDH1A3, ALDH3A2, ALG1, ALG14, ALG2, ALMS1, AMACR, ANGPT1, ANKS6, ANO10, AP3B1, AP3D1, AP5Z1, APC, APOB, APTX, ARFGAP2, ARHGEF18, ARL13B, ARL2, ARL2BP, ARL3, ARL6, ARMC9, ARMS2, ARSG, ASB10, ASIC5, ASPH, ASRGL1, ATAD3A, ATF6, ATOH1, ATOH7, ATP13A2, ATP5F1A, ATP5F1D, ATP5F1E, ATP5MF, ATP5MG, ATP5MGL, ATP5PB, ATP5PD, ATP7B, ATPAF1, ATPAF2, ATXN7, AUH, B3GALNT2, B3GALT6, B3GLCT, B4GALT7, B4GAT1, B9D1, B9D2, BBIP1, BBS1, BBS10, BBS12, BBS18, BBS2, BBS4, BBS5, BBS7, BBS8, BBS9, BCKDHA, BCKDHB, BCO1, BCO2, BCOR, BCS1L, BEST1, BFSP1, BFSP2, BLOC1S3, BLOC1S5, BLOC1S6, BLOC1S8, BLOC2S1, BLOC2S2, BLOC2S3, BLOC3S1, BLOC3S2, BMP4, BMP7, BOLA3, BTD, BUB1B, C12ORF57, C12ORF65, C19ORF12, C1QBP, C1QTNF5, C1R, C2, C21ORF2, C2CD3, C2ORF71, C3, C7orf26, C8A, C8B, C8ORF37, C9, CA4, CA5A, CABP4, CACNA1A, CACNA1F, CACNA2D4, CAPN15, CAPN5, CARS2, CASK, CBS, CC2D2A, CCDC103, CCDC114, CCDC28B, CCDC39, CCDC40, CCER1, CCT2, CD36, CDH23, CDH3, CDHR1, CDK5RAP2, CDKN2A, CEP104, CEP120, CEP164, CEP19, CEP250, CEP290, CEP41, CEP78, CEP83, CERKL, CFAP410, CFB, CFH, CFHR2, CHAT, CHCHD10, CHD7, CHD8, CHERP, CHKB, CHM, CHMP4B, CHN1, CHRNA1, CHRNB1, CHRND, CHRNE, CHST14, CHST6, CIB2, CISD2, CLCC1, CLDN19, CLEC3B, CLN3, CLN5, CLN6, CLN8, CLPB, CLPP, CLRN1, CLTA, CLUAP1, CNGA1, CNGA3, CNGB1, CNGB3, CNNM4, COA3, COA4, COA5, COA6, COA7, COA8, COD2, COL11A1, COL11A2, COL12A1, COL13A1, COL18A1, COL1A1, COL1A2, COL26A1, COL2A1, COL4A1, COL4A3, COL4A4, COL4A5, COL5A1, COL5A2, COL9A1, COL9A2, COL9A3, COLQ, COQ2, COQ4, COQ5, COQ6, COQ7, COQ8A, COQ8B, COQ9, COX10, COX11, COX14, COX15, COX16, COX17, COX18, COX19, COX20, COX4I1, COX4I2, COX6A1, COX6A2, COX6B1, COX6B2, COX7A1, COX7B, COX8A, CPAMD8, CPL3A1, CPLANE1, CPT1A, CPT2, CRB1, CRELD1, CRPPA, CRX, CRYAA, CRYAB, CRYBA1, CRYBA2, CRYBA4, CRYBB1, CRYBB2, CRYBB3, CRYGA, CRYGB, CRYGC, CRYGD, CRYGS, CSMD1, CSMD2, CSPP1, CTDP1, CTNNA1, CTNNB1, CTSD, CTSF, CWC27, CX3CR1, CYC1, CYCS, CYP1B1, CYP27A1, CYP4V2, DAG1, DARS2, DBT, DCC, DCDC1, DCN, DCT, DDX58, DGUOK, DHCR7, DHDDS, DHX32, DHX38, DLAT, DLD, DMD, DNA2, DNAAF1, DNAAF2, DNAAF3, DNAAF5, DNAH11, DNAH5, DNAI1, DNAI2, DNAJC17, DNAJC19, DNAJC30, DNAJC5, DNAL1, DNM1L, DNM2, DOK7, DPAGT1, DRAM2, DSCAML1, DSE, DTHD1, DTNBP1, DYNC2H1, EARS2, ECHS1, EDN3, EDNRB, EFEMP1, ELAC2, ELOVL1, ELOVL4, ELP1, ELP4, EMC1, ENSA, EPHA2, ERAL1, ERCC6, ERCC8, ESCO2, ESPN, ETFA, ETFB, ETFDH, ETHE1, EVC, EVC2, EXOSC2, EYA1, EYS, FA2H, FAM126A, FAM161A, FARS2, FASTKD2, FBLN5, FBN1, FBP1, FBXL4, FCN1, FDFT1, FDX2, FDXR, FGF21, FH, FKBP14, FKRP, FKTN, FLAD1, FLNB, FLVCR1, FOXC1, FOXC2, FOXE3, FOXH1, FOXRED1, FRAS1, FREM2, FRMD7, FSCN2, FTL, FXN, FYCO1, FZD4, FZD5, G6PC, GAA, GALE, GALK1, GALM, GALT, GAMT, GARS1, GATB, GATC, GATM, GCDH, GCNT2, GDAP1, GDF1, GDF3, GDF6, GFER, GFM1, GFM2, GFPT1, GJA1, GJA3, GJA8, GJB1, GJB2, GJB6, GJC3, GLIS2, GLRX5, GMPPB, GNAT1, GNAT2, GNB3, GNPAT, GNPTG, GNS, GPR125, GPR143, GPR179, GPR45, GRIP1, GRK1, GRM6, GRN, GTPBP3, GUCA1A, GUCA1B, GUCA1C, GUCY2D, GYS2, GZF1, HADH, HADHA, HADHB, HARS, HARS1, HARS2, HCCS, HCN1, HESX1, HGSNAT, HIBCH, HK1, HKDC1, HLCS, HMCN1, HMGB3, HMGCL, HMGCS2, HMX1, HPS1, HPS3, HPS4, HPS5, HPS6, HSD17B10, HSF4, HSPD1, HTRA1, HTRA2, HYLS1, IARS1, IARS2, IBA57, IDH3A, IDH3B, IDUA, IFT122, IFT139, IFT140, IFT144, IFT172, IFT27, IFT38, IFT43, IFT52, IFT54, IFT74, IFT80, IFT81, IFT88, IMPDH1, IMPG1, IMPG2, INPP5E, INTS1, INVS, IQCB1, ISCA1, ISCA2, ISCU, ITM2B, JAG1, JAM3, KARS1, KCNJ13, KCNV2, KCTD7, KERA, KIAA0556, KIAA0586, KIAA1549, KIF11, KIF24, KIF3B, KIF7, KIZ, KLHL7, LAMA1, LAMA5, LAMB2, LAMP2, LARGE1, LARS1, LARS2, LCA5, LCT, LDLR, LEFTY2, LHX2, LIAS, LIM2, LIPT1, LIPT2, LMX1B, LONP1, LOXL1, LOXL3, LRAT, LRIT3, LRMDA, LRP2, LRP4, LRP5, LRPPRC, LRRC6, LTBP2, LTBP3, LYRM4, LYRM7, LYST, LZTFL1, MAB21L1, MAB21L2, MACF1, MAF, MAK, MAN2B1, MAPKAPK3, MAPKBP1, MARS2, MASP1, MASP2, MDH2, MECR, MERTK, MFF, MFN2, MFRP, MFSD8, MGME1, MICOS13, MICU1, MIEF1, MIP, MIPEP, MIR204, MITF, Mitochondria genome, MKKS, MKS1, MLPH, MMACHC, MMP1, MPC1, MPRIP, MPV17, MRM2, MRPL12, MRPL3, MRPL44, MRPS14, MRPS16, MRPS2, MRPS22, MRPS23, MRPS34, MRPS7, MRRF, MRS2, MSTO1, MTFMT, MTO1, MTPAP, MTTP, MUSK, MVK, MYH9, MYO5A, MYO7A, MYO9A, MYOC, NAA10, NADK2, NARS2, NAXE, NBAS, NDP, NDRG4, NDUFA1, NDUFA10, NDUFA11, NDUFA12, NDUFA13, NDUFA2, NDUFA4, NDUFA6, NDUFA7, NDUFA8, NDUFA9, NDUFAF1, NDUFAF2, NDUFAF3, NDUFAF4, NDUFAF5, NDUFAF6, NDUFAF7, NDUFAF8, NDUFB11, NDUFB3, NDUFB6, NDUFB8, NDUFB9, NDUFS1, NDUFS2, NDUFS3, NDUFS4, NDUFS5, NDUFS6, NDUFS7, NDUFS8, NDUFV1, NDUFV2, NDUFV3, NECTIN3, NEFH, NEK1, NEK2, NEK8, NEUROD1, NF2, NFS1, NFU1, NHS, NKX2-5, NME8, NMNAT1, NOD2, NODAL, NOTCH3, NPHP1, NPHP3, NPHP4, NR2E3, NR2F1, NRL, NSUN3, NTF4, NUBPL, NUTF2, NXNL1, NYX, OAT, OCA2, OCRL, OFD1, OPA1, OPA3, OPA5, OPN1LW, OPN1MW, OPN1SW, OPTN, OR2W3, OTC, OTOGL, OTX2, OXA1L, P3H2, PANK2, PARS2, PAX2, PAX3, PAX6, PC, PCARE, PCCA, PCCB, PCDH15, PCK2, PCYT1A, PDE6A, PDE6B, PDE6C, PDE6D, PDE6G, PDE6H, PDHA1, PDHB, PDHX, PDP1, PDSS1, PDSS2, PDX1, PDZD7, PET100, PET117, PEX1, PEX10, PEX11B, PEX12, PEX13, PEX14, PEX16, PEX19, PEX2, PEX26, PEX3, PEX5, PEX6, PEX7, PGK1, PHYH, PIBF1, PITPNM3, PITRM1, PITX2, PITX3, PKM, PLA2G5, PLA2G6, PLEC, PLK4, PLOD1, PLOD3, PMP22, PMPCA, PMPCB, PNPLA6, PNPLA8, PNPT1, POC1B, POC5, POLG, POLG2, POLRMT, POMGNT1, POMGNT2, POMK, POMT1, POMT2, PORCN, PPA2, PPT1, PRCD, PRDM13, PRDM5, PREPL, PROM1, PROS1, PRPF3, PRPF31, PRPF4, PRPF6, PRPF8, PRPH2, PRPS1, PRR12, PRSS56, PTCD1, PTCD3, PTCH1, PUS1, PXDN, PYGM, QARS1, QRSL1, RAB18, RAB27A, RAB28, RAB3GAP1, RAB3GAP2, RAPSN, RARB, RARS1, RARS2, RAX, RAX2, RB1, RBP3, RBP4, RCBTB1, RCD1, RD3, RDH11, RDH12, RDH5, RECQL4, REEP1, REEP6, RERE, RGR, RGS9, RGS9BP, RHEX, RHO, RIMS1, RIMS2, RLBP1, RMND1, RNANC, RNASEH1, ROM1, RP1, RP1L1, RP2, RP9, RPE65, RPGR, RPGRIP1, RPGRIP1L, RPH3A, RPL10, RPL15, RRM2B, RS1, RSPH4A, RSPH9, RTBDN, RTN4IP1, SACS, SAG, SALL2, SAMD11, SARS2, SBF2, SC5D, SCAPER, SCARF2, SCLT1, SCN4A, SCO1, SCO2, SDCCAG8, SDHA, SDHAF1, SDHAF2, SDHAF3, SDHAF4, SDHB, SDHC, SDHD, SEC23A, SEMA4A, SERAC1, SETX, SFXN4, SHH, SIL1, SIX3, SIX5, SIX6, SLC16A12, SLC18A3, SLC19A2, SLC19A3, SLC22A5, SLC24A1, SLC24A5, SLC25A1, SLC25A12, SLC25A13, SLC25A15, SLC25A19, SLC25A20, SLC25A21, SLC25A26, SLC25A3, SLC25A32, SLC25A38, SLC25A4, SLC25A42, SLC25A46, SLC2A1, SLC33A1, SLC37A4, SLC38A8, SLC39A13, SLC45A2, SLC4A7, SLC52A2, SLC52A2, SLC5A7, SLC6A15, SLC6A8, SLC7A14, SMOC1, SNAI2, SNAP25, SNRNP200, SNX10, SNX3, SOD1, SOX10, SOX2, SOX3, SPAST, SPATA5, SPATA7, SPG11, SPG15, SPG2, SPG35, SPG45, SPG46, SPG54, SPG55, SPG57, SPG68, SPG7, SPG9, SPP2, SRD5A3, SREBF2, SSBP1, STARD9, STAT2, STK38L, STRA6, SUCLA2, SUCLG1, SUFU, SUOX, SURF1, SYNE2, SYT2, SYTL4, TACO1, TARS2, TAZ, TBC1D20, TBC1D32, TBK1, TCTN1, TCTN2, TCTN3, TDRD7, TEAD1, TEK, TENM3, TFAM, TFAP2A, TFB1M, TGFBI, TIE2, TIMM22, TIMM50, TIMM8A, TIMMDC1, TIMP3, TK2, TKFC, TLCD3B, TLR3, TLR4, TMED7, TMEM107, TMEM114, TMEM126A, TMEM126B, TMEM138, TMEM216, TMEM218, TMEM231, TMEM237, TMEM65, TMEM67, TMEM70, TMEM98, TNPO1, TNXB, TOP3A, TOPORS, TOR1AIP1, TPK1, TPP1, TRAF7, TREX1, TRIM32, TRIT1, TRMT10C, TRMT5, TRMU, TRNT1, TRPM1, TSFM, TSPAN12, TTC19, TTC21B, TTC8, TTLL5, TTPA, TUB, TUBB4B, TUBGCP4, TUBGCP6, TUFM, TULP1, TWNK, TYMP, TYR, TYRP1, UBIAD1, UCHL1, UNC119, UNC13A, UQCC1, UQCC2, UQCC3, UQCR10, UQCR11, UQCRB, UQCRC2, UQCRQ, USH1C, USH1G, USH2A, USH2C, USH2D, VAMP1, VARS2, VAX1, VAX2, VCAN, VPS13B, VSX2, WARS2, WDPCP, WDR17, WDR19, WDR34, WDR35, WDR36, WFS1, WFS2, WHRN, XPNPEP3, YARS2, YME1L1, ZEB2, ZIC3, ZNF408, ZNF423, ZNF469, ZNF513, ZNHIT3.

LIMITATIONS: Only the coding regions and immediately flanking intron sequences were examined. Changes in the promoter region, deep intronic regions, or other non-coding regions of the gene would not be detected. The sensitivity of DNA sequencing is 99% for the detection of nucleotide base changes, small deletions and insertions in the regions analyzed. Multiple exon deletions, multiple exon insertions, and complete deletion of one allele may not be identified using these methods. NGS CNV analysis was also performed. However, smaller deletion and duplications may not be identified by the method.

3B-EXOME

METHODS

Extracted DNA was received for testing. Exome capture was performed using xGen Exome Research Panel v2, supplemented with xGen human mtDNA panel and xGen Custom Hyb Panel v2 (Integrated DNA Technologies, Coralville, Iowa, USA). Sequencing was performed using NovaSeq X (Illumina, San Diego, CA, USA). In total, 12,060,804,170 bases of sequence were generated and uniquely aligned to a modified version of the Genome Reference Consortium Human Build 38 (GRCh38), in which falsely duplicated regions of chromosome 21 were masked to N's based on the masking file developed in collaboration with the GRC (Genome Reference Consortium) (Nat Biotechnol. 2022;40:672-680) and Revised Cambridge Reference Sequence (rCRS) of the mitochondrial genome, generating 174.35 mean depth-of-coverage within the 35,392,153 bases of the captured region, which is approximately 99.3% of the RefSeq protein coding region. Approximately 98.70% of the targeted bases were covered to a depth of ≥20x. Despite the insufficient coverage across 1.30% of the bases (see below for details), these metrics are consistent with high quality exome sequencing data and deemed adequate for analysis. Gene or exon level depth-of-coverage (DOC) information is available upon request. In total, 64,788 single nucleotide variants (SNV) and 12,363 small insertions and deletions (INDEL) were identified. Sequencing data analysis and variant interpretation were performed using 3billion's proprietary system, EVIDENCE v4.3 (Clin Genet. 2020;98:562-570). EVIDENCE incorporates bioinformatics pipeline for calling SNV/INDEL based on the GATK best practices (GATK v4.4.0, Genome Res. 2010;20:1297–303) and Manta v1.6.0 (Bioinformatics. 2016;32:1220-2) for calling CNV (copy number variants) based on paired-end information and 3bCNV v2.1, an internally developed tool, for calling CNV (copy number variants) including aneuploidy based on the DOC information. It also incorporates Mutect2 v4.4.0 (Genome Res. 2010;20:1297–303) for calling lower level heteroplasmic SNV/INDEL in the mitochondrial genome, ExpansionHunter v5.0.0 (Bioinformatics. 2019;35:4754–6) for calling repeat expansion variants, MELT v2.2.2 (Genome Res. 2017;27:1916–29) for calling mobile element insertion variants, AutoMap v1.3 (Nat Commun. 2021;12:518) for detecting regions of homozygosity (ROH). Variant Effect Predictor v112.0 (VEP, Ensembl, Genome Biology 2016;17:122) is used for variant annotation. Variants were prioritized based on the guideline recommended by the American College of Medical Genetics and Genomics (ACMG) and the Association for Molecular Pathology (AMP) (Genet Med. 2015;17:405-424, Genet Med. 2020:22:245-257, and Hum Mutat. 2020;41:2028-2057) in the context of the patient's phenotype, relevant family history and previous test results provided by the ordering physician. Only variants deemed clinically significant and relevant to the patient's clinical indications at the time of variant interpretation are reported. Based on internal studies validating the accuracy of the variants called with high quality scores, only low quality variants are confirmed by Sanger sequencing. The raw data files including FASTQ files, VCF files and/or annotated small [illegible]

DISCLAIMER

This test was developed by 3billion in the purpose of identifying single nucleotide variants (SNV), small insertions/deletions (INDEL, <50 bp), large (>=3 consecutive exons) copy number variants, mobile element insertion variants and repeat expansion variants within the targeted genomic regions. Repeat expansion detection is possible for the following 18 genes. Repeat expansion number may be underestimated for the starred (*) gene with compromised sensitivity (*AR, ARX, ATN1, ATXN1, ATXN2, ATXN3, ATXN7, ATXN8OS*, CACNA1A, COMP, FOXL2, HOXD13, HTT, PABPN1, PHOX2B, PRDM12, TBP, ZIC2*). Only SNV/INDEL (>10% heteroplasmic level) are called within the mitochondrial genome. This test is intended for clinical purposes and should not be regarded as investigational or for research. This laboratory is certified under the College of American Pathologists (CAP#:8750906) and Clinical Laboratory Improvement Amendments (CLIA#: 99D2274041) as qualified to perform high complexity clinical laboratory testing. Assay validation and clinical validation were performed following the Korea Institute of Genetic Testing Evaluation and the American College of Medical Genetics and Genomics (ACMG) Technical Standards and Guidelines Section G (https://www.acmg.net/PDFLibrary/Standards-Guidelines-Clinical-Molecular-Genetics.pdf). If other types of variants such as translocation, inversion, low-level mosaicism, low heteroplasmic level mitochondrial genome variants, and mitochondrial genome large deletion/duplication are suspected, it is recommended to perform appropriate testing that are designed to detect those types of variants. Also, there are certain exonic regions that are incompletely sequenced due to technical difficulties with amplification, sequencing and alignment. If variants within these regions are suspected, it is recommended to perform alternate testing that are designed to sequence those regions/genes adequately. This report may not be copied or reproduced, except in its totality.

ACCREDITATIONS AND CERTIFICATIONS

CAP License #
8750906, AU-ID# 2052626

CLIA ID #
99D2274041

PRIMARY FINDINGS INTERPRETATION

GNAT1 NM_144499.3:c.599A>G (NP_653082.1:p.Gln200Arg)	
**Population Data**	The variant is not observed in the gnomAD v4.1.0 dataset.
**Predicted Consequence/Location**	Missense variant
**Segregation Data**	None
**Computation and Functional Data**	*In silico* tool predictions suggest damaging effect of the variant on gene or gene product [REVEL: 0.94 (>=0.6, sensitivity 0.68 and specificity 0.92); 3Cnet: 0.95 (> 0.75, sensitivity 0.96 and precision 0.92)].
**Previously Reported Variant Data**	A different missense change at the same codon (p.Gln200Glu) has been reported to be associated with *GNAT1*-related disorder (ClinVar ID: VCV000190967 / PMID: 17584859).
**Disease Association**	Night blindness, congenital stationary, autosomal dominant 3 (OMIM: 610444)
**Validation**	Not performed as the variant was considered high-quality
**Variant Classification**	VUS

ADDITIONAL FINDINGS

No additional variants were identified, including variants of uncertain significance (VUSs) that could not be reported as primary findings due to limited evidence of pathogenicity, even though they may explain the patient's symptoms; pathogenic, likely pathogenic variants or VUSs that may partially explain the patient's symptoms, regardless of whether they fit the mode of inheritance; or variants associated with the family history provided by the healthcare provider, regardless of the patient's current symptoms.

SECONDARY FINDINGS

Receiving the secondary findings was opted out by the patient. Consult with the medical provider.

Chromatograms

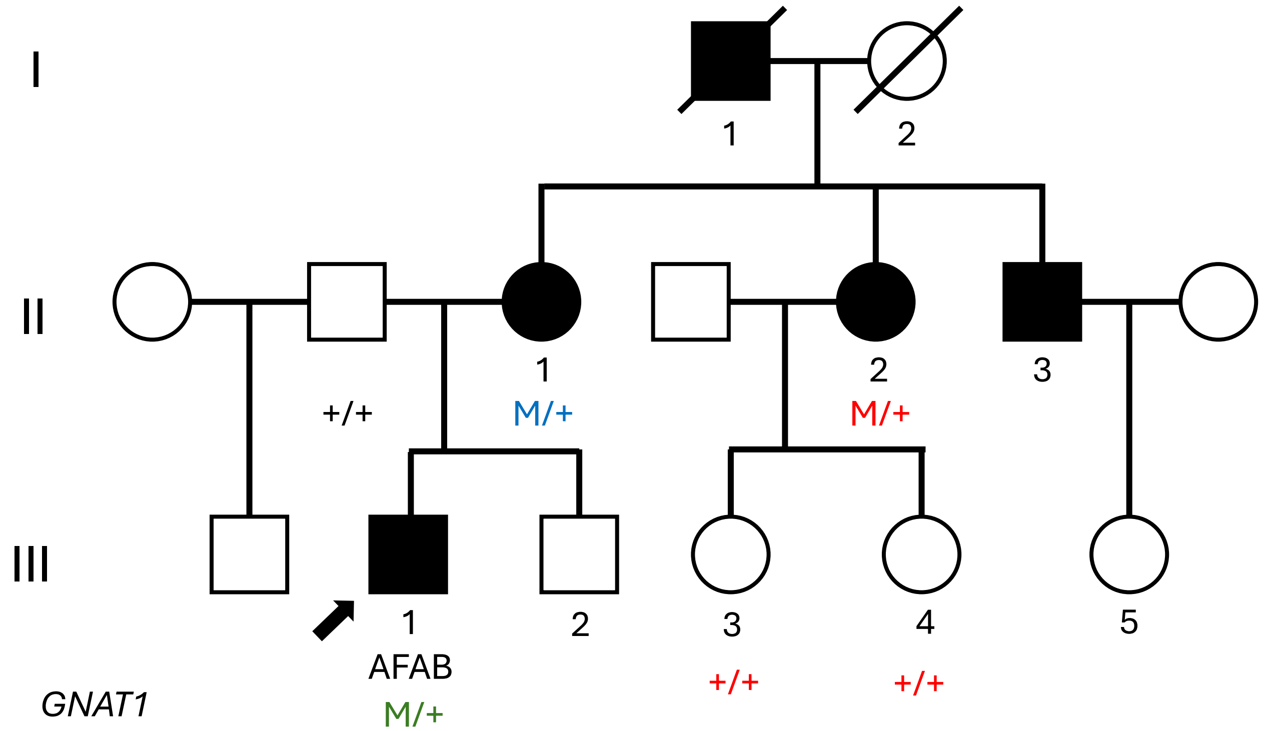

GNAT1
M: c.599A>G
+: no variant
AFAB: assigned female at birth

MVL next generation sequencing (MVL Vision Panel)

3 billion whole exome sequencing (3B-EXOME)

MVL Sanger Sequencing

In-house Sanger Sequencing: Chromatograms shown below

II:2 (IRD9146)
c.599A>G;599A=

Forward Primer Sequencing:

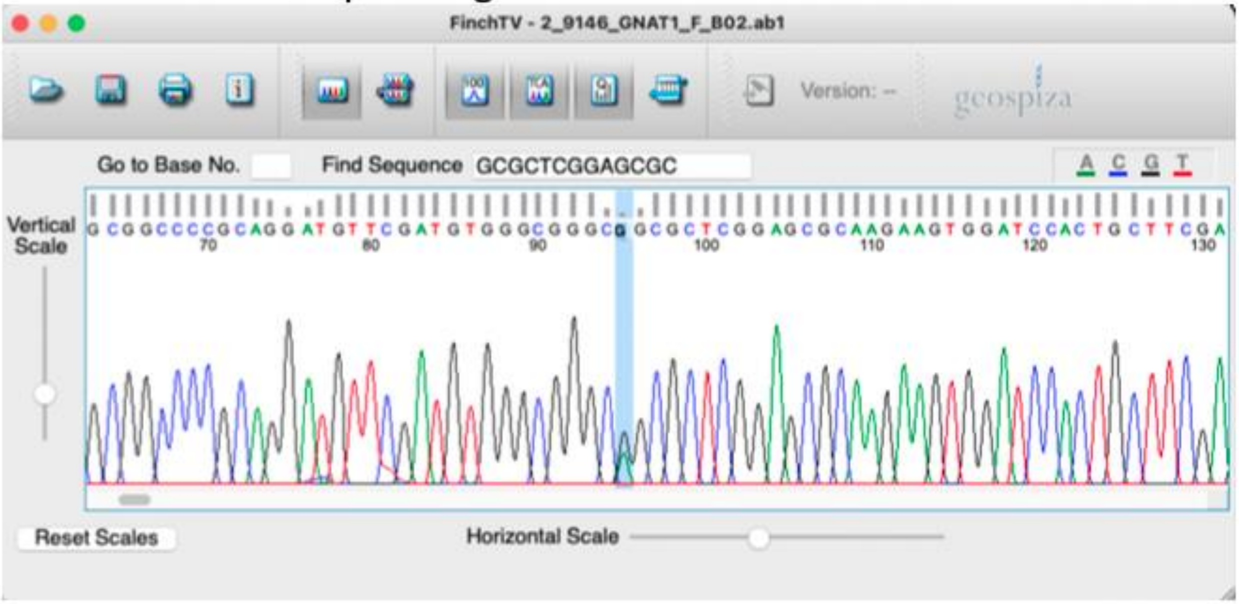

Reverse Primer Sequencing:

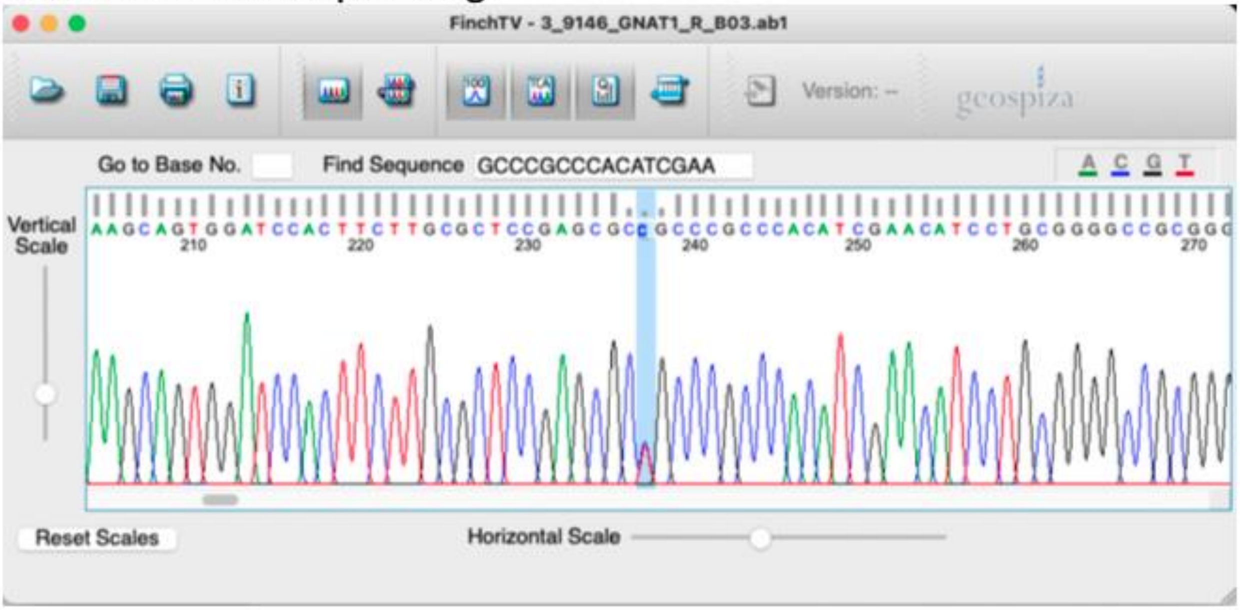

III:3 (IRD9147)
c.599A=;599A=

IRD9147 Forward Primer Sequencing:

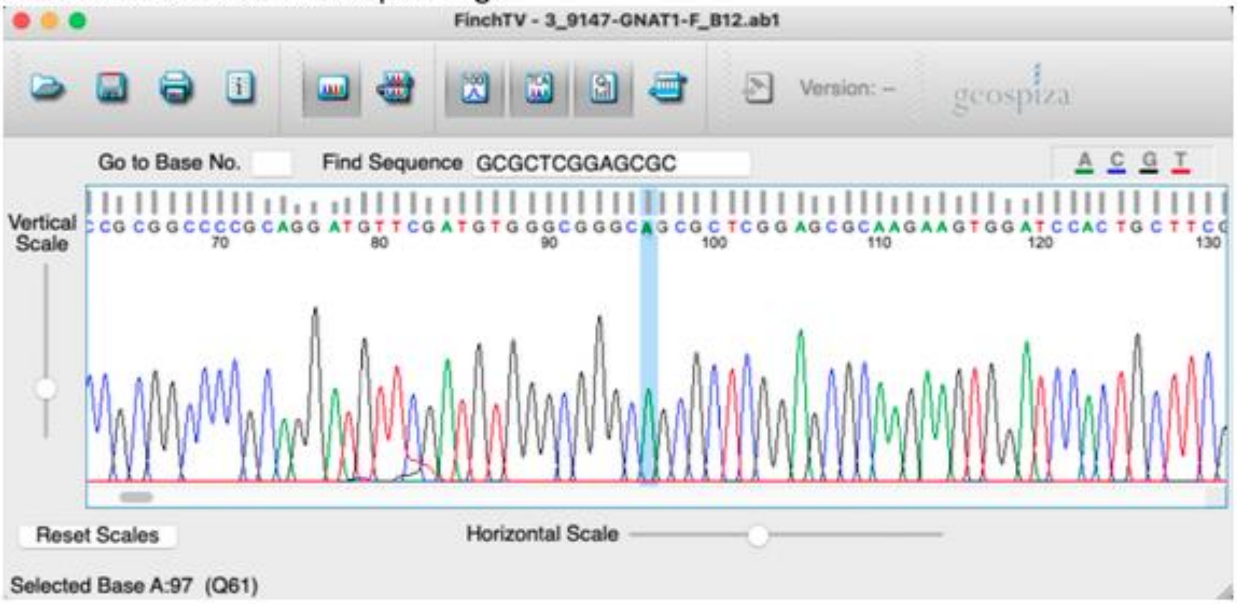

IRD9147 Reverse Primer Sequencing:

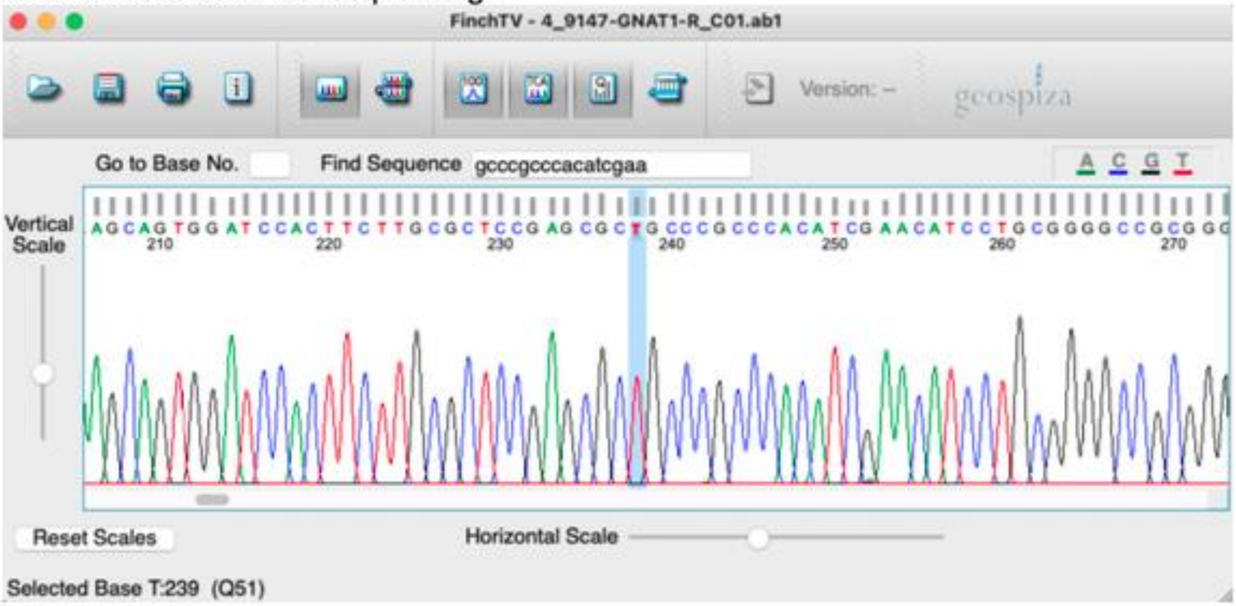

III:4 (IRD9145)
c.599A=;599A=

IRD9145 Forward Primer Sequencing:

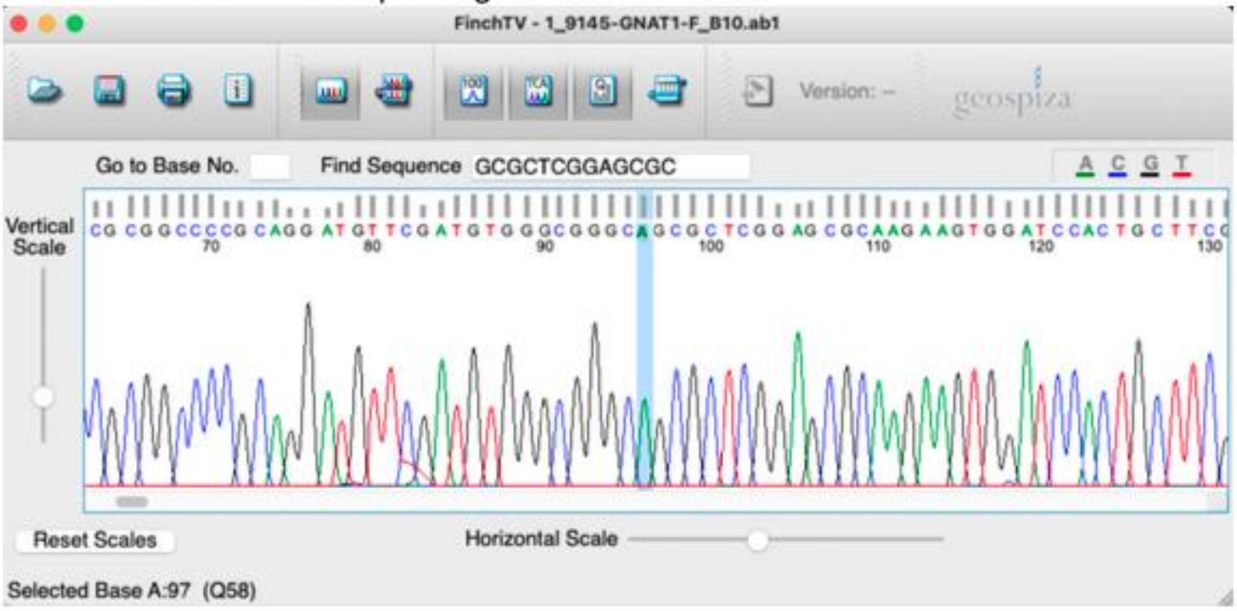

IRD9145 Reverse Primer Sequencing:

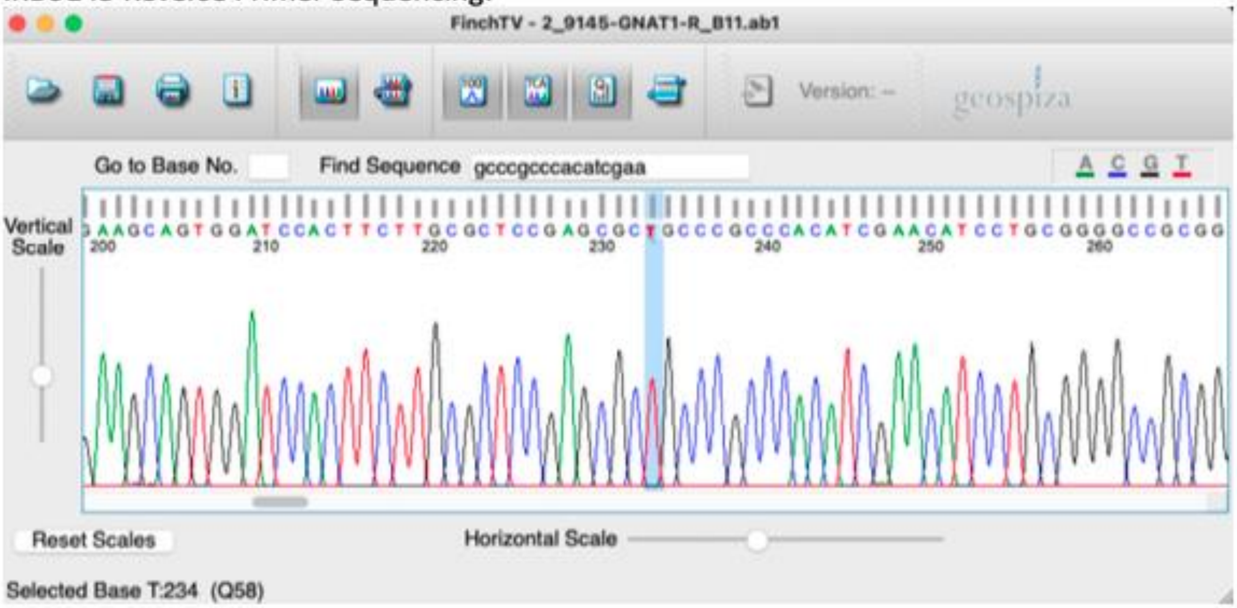

Supplement: Supplementary file 1 — Supplementary file1 (PDF 958 KB) [file 10633_2026_10100_MOESM1_ESM.pdf]
